# Clinical Factors Associated with Sperm DNA Fragmentation in Male Patients with Infertility

**DOI:** 10.1155/2014/868303

**Published:** 2014-08-04

**Authors:** Akira Komiya, Tomonori Kato, Yoko Kawauchi, Akihiko Watanabe, Hideki Fuse

**Affiliations:** Department of Urology, Graduate School of Medicine and Pharmaceutical Sciences for Research, University of Toyama, 2630 Sugitani, Toyama-shi, Toyama 930-0194, Japan

## Abstract

*Objective*. The clinical factors associated with sperm DNA fragmentation (SDF) were investigated in male patients with infertility. *Materials and Methods*. Fifty-four ejaculates from infertile Japanese males were used. Thirty-three and twenty-one were from the patients with varicoceles and idiopathic causes of infertility, respectively. We performed blood tests, including the serum sex hormone levels, and conventional and computer-assisted semen analyses. The sperm nuclear vacuolization (SNV) was evaluated using a high-magnification microscope. The SDF was evaluated using the sperm chromatin dispersion test (SCDt) to determine the SDF index (SDFI). The SDFI was compared with semen parameters and other clinical variables, including lifestyle factors. *Results*. The SDFI was 41.3 ± 22.2% (mean ± standard deviation) and did not depend on the cause of infertility. Chronic alcohol use increased the SDFI to 49.6 ± 23.3% compared with 33.9 ± 18.0% in nondrinkers. The SDFI was related to adverse conventional semen parameters and sperm motion characteristics and correlated with the serum FSH level. The SNV showed a tendency to increase with the SDFI. The multivariate analysis revealed that the sperm progressive motility and chronic alcohol use were significant predictors of the SDF. *Conclusion*. The SCDt should be offered to chronic alcohol users and those with decreased sperm progressive motility.

## 1. Introduction

A semen analysis remains the main tool for evaluation of male infertility [1, 2]. The conventional microscopic examination of semen is simple and inexpensive but used to be prone to high variability [3]. In the treatment of male infertility, the parameters of the conventional semen analysis do not reliably predict either male fertility or the likelihood of pregnancy after infertility treatment. Thus, researchers have sought methods to predict male fertility in a more clinically useful manner [4]. In this context, we have previously demonstrated the clinical application of the sperm motility analysis system (SMAS) as one of computer-assisted semen analysis systems and the observation of sperm nuclear vacuoles using a high-magnification microscope in male patients with infertility [3, 5]. Large sperm nuclear vacuoles are thought to be related not only to poor ART outcomes but also to poor semen quality and sperm DNA damage, such as DNA fragmentation and chromatin condensation failure [6, 7].

In addition to these parameters, the sperm DNA fragmentation is being increasingly recognized as an important cause of infertility and is being widely investigated. The association between DNA damage and diminished reproductive outcomes has led to the introduction of sperm DNA integrity testing to the clinical assessment of male fertility [4]. The integrity of the sperm DNA is essential for the accurate transmission of genetic information. Any form of sperm chromatin abnormalities or DNA damage may result in male infertility [8]. The most commonly performed DNA integrity tests are the sperm chromatin structure assay (SCSA) [9], the deoxynucleotidyl transferase-mediated dUTP nick end labeling assay (TUNEL) [10], the single-cell gel electrophoresis assay (Comet) [11], and, most recently, the sperm chromatin dispersion test (SCDt) [4, 12]. Numerous studies utilizing these techniques to assess the sperm DNA integrity have supported the existence of a significant association between sperm DNA damage and pregnancy outcomes in humans [13].

These methods are considered to be an independent measure of sperm quality that may yield better diagnostic and prognostic approaches than standard sperm parameters (concentration, motility, and morphology) [8]. A guideline for the clinical utility of sperm DNA integrity testing is now available [4]. However, the rates of spermatozoa with DNA damage vary among studies, possibly due to the study populations and the methods used for DNA integrity testing [14–16]. In addition, there have been few studies that have investigated the correlation between the clinical factors and sperm DNA damage. Among the modifiable lifestyle factors, smoking is thought to induce sperm DNA/genetic damage and cause poor semen quality [17, 18]. Associations between alcohol exposure and reduced male fertility have also been the subjects of various studies [19]. Alcohol exposure causes alterations of the endocrine system controlling the hypothalamic-pituitary-testicular axis function and a direct toxic effect on testis and/or male accessory glands [20–22]. Alcohol exposure is also reported to influence sperm DNA integrity [23, 24].

The SCDt is based on the principle that sperm with fragmented DNA fails to produce the characteristic halo of dispersed DNA loops that are observed in sperm with nonfragmented DNA, following acid denaturation and removal of nuclear proteins. The SCDt distinguishes cells with intact DNA (large halo) from sperm cells with damaged DNA (small or absent halo). Among the DNA integrity tests mentioned above, the TUNEL and SCSA are the most commonly used so far; however, the SCDt was introduced as a simple, fast, accurate, and highly reproducible method for the analysis of sperm DNA fragmentation in semen and processed sperm. In addition, the SCDt does not require the use of complex instrumentation; it can be carried out with equipment normally available in andrology laboratories (i.e., light microscopes), and the test endpoints (nondispersed and dispersed nuclei) can be easily assessed by laboratory technicians [12, 25].

In the present study, we evaluated the sperm DNA fragmentation measured by SCDt in male Japanese patients with infertility. The relationships between the sperm DNA fragmentation index (SDFI) and clinical parameters such as age, smoking status, alcohol drinking status, serum sex hormone levels, causes of male infertility, conventional semen parameters, sperm motility parameters measured by SMAS, and sperm nuclear vacuolization observed by a high-magnification microscope were evaluated to determine which factors influence the sperm DNA fragmentation in this cohort.

## 2. Materials and Methods

The Institutional Review Board of the University of Toyama approved this study (#23-128). Ethical consent for the work to be carried out was provided, and signed informed consent was obtained from each patient evaluated in this study. The study conformed to the principles outlined in the Declaration of Helsinki.

### 2.1. Sample Collection

We used 54 ejaculates from male Japanese patients with infertility who visited the Male Infertility Clinic at Toyama University Hospital between October 2012 and February 2014. These patients underwent a conventional semen analysis, computer-assisted semen analysis by the SMAS, high-magnification observation of the sperm heads, and sperm DNA integrity testing during the evaluation for male infertility. Medical treatment by Japanese herbal medicine was used prior to semen analysis in 8 patients for 5.6 months in average (2 to 14). The patients were asked for at least five days of abstinence before semen analyses. The semen samples were collected following masturbation, were allowed to liquefy at room temperature, and were evaluated within one hour of collection using the manual conventional semen analyses, which were performed as described previously [3, 5]. All manual assessments were performed by a single experienced laboratory technician (Y.K.), and the sperm concentrations were assessed using an improved Neubauer hemocytometer. The samples were diluted according to the instructions in the WHO laboratory manual (1999) [26].

To determine the degree of sperm motility, a 10 *μ*L sample was loaded onto a clear slide glass and covered with a 22 × 22 mm^2^ cover glass under a positive phase-contrast microscope at a total magnification of ×400. The definition of male infertility included the failure to conceive following twelve months of unprotected intercourse due to possible male factors. At least one parameter of a conventional semen analysis was abnormal in these patients. Male factors were generally screened based on a medical history, physical examinations, conventional semen analyses, and blood tests, including assessments of pituitary and sex hormones (luteinizing hormone, follicle-stimulating hormone, and testosterone). We asked about the patients' smoking and alcohol drinking status. Chronic alcohol use was defined by the consumption of ≥350 mL of beer per week or a corresponding amount of other alcoholic-containing drinks. The serum follicle-stimulating hormone and luteinizing hormone levels were measured by chemiluminescent immunoassays. The serum total testosterone concentration was measured by electrochemiluminescent immunoassays. The serum free testosterone concentration was measured by solid phase radioimmunoassays. Varicoceles were diagnosed during scrotal examinations with the patient in a standing position and were graded as described previously [27]. Those who showed a sperm count less than 5 million/mL were excluded, because the SCDt requires a concentration of 5–10 million/mL. The Two-Step Discontinuous PureCeption Gradient Technique was used to select motile spermatozoa according to the manufacturer's manual for the PureCeption Determination Kits (Nakamedical, Tokyo, Japan), as described previously [5]. Processed spermatozoa were used in the observation by a high-magnification microscope and the sperm chromatin dispersion test.

### 2.2. Computer-Assisted Semen Analysis (CASA) Using the Sperm Motility Analysis System (SMAS)

The SMAS consisted of a digital scanning camera, a personal computer with a digital frame grabber with image processing software, and a monitor. This system records images at 60 frames per second and can analyze up to approximately 200 spermatozoa simultaneously in real time. Similar to the conventional CASA system, the SMAS can analyze sperm motion parameters such as the linear velocity, curvilinear velocity, linearity, amplitude of lateral head displacement (ALH), and beat-cross frequency (BCF), in addition to the percent motility and sperm concentration. Furthermore, the performance of the SMAS can be monitored at any time by comparing SMAS-determined parameters with manually determined values derived from the same image, which is overlaid with colored lines showing the motion paths of the spermatozoa. The most successful image analysis of spermatozoa is obtained with a positive phase-contrast microscope that can be used by selecting the optimum light intensity. For each measurement, a 10 *μ*L aliquot was loaded into a Makler cell counting chamber (Seifi-Medical Instruments, Haifa, Israel) [3].

### 2.3. Observation of Spermatozoa by a High-Magnification Microscope

**Figure 1:**
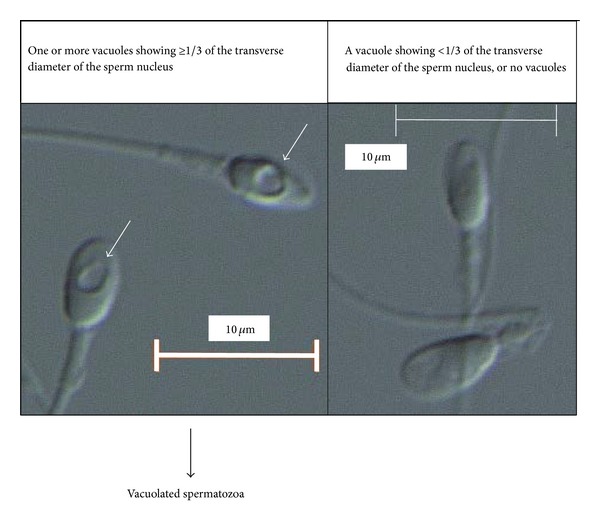
A high magnification view obtained using an inverted microscope equipped with Nomarski differential interference contrast optics and a video system. The arrows indicate nuclear vacuoles observed through a 60x (1.42 numerical aperture) objective lens.

The selected spermatozoa were analyzed at 3700x magnification using an inverted microscope equipped with Nomarski differential interference contrast optics (IX71, Olympus, Tokyo) and a video system (FX630, Olympus, Tokyo). A 60x (1.42 numerical aperture) objective lens was used with oil. The images of the spermatozoa were captured and stored using an image-filing software program, FlvFs (Flovel, Tokyo), on a video system. We spent 30 to 60 minutes capturing and analyzing the images of each ejaculate. Originally, 100 spermatozoa per ejaculate were evaluated using a motile sperm organelle morphology examination (MSOME) [28, 29]; however, this was not always possible due to the poor semen quality in this cohort. A spermatozoon was defined as “vacuolated” if the maximum diameter of the sperm nuclear vacuole was more than one-third of the width of the sperm head (Figure 1). This definition is slightly modified from that used in our previous report [5].

### 2.4. Sperm Chromatin Dispersion Test

The sperm DNA fragmentation was evaluated by SCDt according to the manufacturer's instructions for the Halosperm kit (Halotech Dna, Madrid, Spain) as follows: the processed semen sample was diluted in phosphate buffer solution (pH 6.88) to a concentration of 5–10 × 10^6^/mL. The agarose eppendorf provided in the kit was put through a float and left in water for five minutes at 90–100°C until the agarose dissolved. Then, the agarose eppendorf was transferred to a temperature controlled water bath maintained at 37°C and left for five minutes until the temperature was even throughout the tube. A total of 25 *μ*L of the semen sample was added to the agarose eppendorf and mixed well. A 14 *μ*L aliquot of the cell suspension was placed from the agarose eppendorf on the treated side of the glass slide provided in the kit and was covered with an 18 × 18 mm^2^ glass coverslip, avoiding the formation of air bubbles. The slide was kept in a horizontal position throughout the entire process. Then, the slide was placed on a cold surface at 4°C and left for five minutes. The cover slide was removed by sliding it off gently. The slide was immersed in the acid denaturation solution provided in the kit and left to incubate at room temperature for 7 minutes horizontally. Afterwards, the slide was placed in another incubation tray containing 10 mL of tempered lysis solution provided in the kit to incubate at room temperature for 25 minutes. The slide was then transferred to another incubation tray containing abundant distilled water in order to wash out the lysis solution and was left to incubate for five minutes. The slide was placed into a tray containing 70% ethanol for two minutes, followed by 90% ethanol for two minutes, and, finally, 100% ethanol for two minutes. The slide was left to dry at room temperature and stained by Wright stain solution (Sigma-Aldrich, Tokyo, Japan).

**Figure 2:**
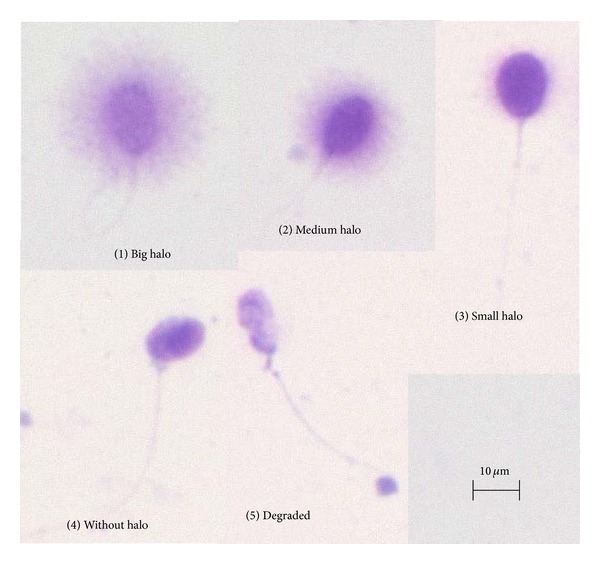
The evaluation of the sperm DNA fragmentation using Halosperm with a bright field microscope through a 20x optical lens. (1)-(2) Spermatozoa without DNA fragmentation. (3)–(5) Spermatozoa with DNA fragmentation.

More than 500 spermatozoa for each ejaculate were evaluated for the sperm DNA fragmentation to calculate the SDFI, which was determined using the following formula: SDFI (%) = 100 × (number of spermatozoa with fragmented DNA)/(number of spermatozoa counted). Spermatozoa with large or medium halos were considered to be free from sperm DNA fragmentation. On the other hand, spermatozoa with small or no halos were considered to be with sperm with DNA fragmentation. Degraded sperm was also considered to be with sperm DNA fragmentation (Figure 2).

### 2.5. Statistical Analysis

The statistical analysis of the data was carried out using the JMP 8.0.1 statistical software package (SAS Institute Japan, Tokyo). Student's *t*-tests were used to compare the values between the groups. Spearman's rank correlation coefficient was used to determine the correlations between the SDFI and the clinical parameters. As a multivariate analysis, predictive variables, which were divided into two groups based on the median values, were evaluated by multiple linear regressions to predict sperm DNA fragmentation. The data are presented as medians with interquartile ranges (IQR) and/or mean values ± standard deviations (S.D.). A value of *P* < 0.05 was defined as being statistically significant.

## 3. Results

### 3.1. The Patients' Characteristics and Results of the Semen Analyses

Fifty-four showing sperm count ≥500 million/mL are included in the analyses. The mean age of the patients and their female partners was 35 and 33 years old, respectively. The mean duration of infertility was 35 months. Out of 54 semen samples, 21 (38.9%) were from the male patients with idiopathic causes of infertility. The remaining 33 (61.1%) were from those with varicoceles (11 from grade II varicocele and 22 from grade III one, Table 1). Twenty-seven (50%) were from the patients who reported chronic alcohol use to some extent. Ten (18.5%) were from current smokers. Based on the conventional semen analyses, the mean values of normal sperm morphology, the sperm count, and the progressive sperm motility were 2.1%, 49.8 × 10^6^/mL, and 33.3%, respectively. Based on the computer-assisted semen analyses using the SMAS, the mean values of the linear velocity, curvilinear velocity, linearity, ALH, and BCF were 17.8 *μ*m/sec, 45.0 *μ*m/sec, 0.40, 1.048 *μ*m, and 11.19 Hz, respectively. High-magnification observations of the sperm heads revealed that the mean proportion of vacuolated spermatozoa was 24.7% using the processed semen samples (Table 2 and Figure 1).

### 3.2. Sperm DNA Fragmentation Index

The mean SDFI evaluated by SCDt using processed semen samples was 41.3% in this cohort (Table 2 and Figure 2). The SDFI was not related to the causes of male infertility; the mean SDFI was 41.3% in both those with idiopathic causes of infertility and those with palpable varicoceles. The SDFI was also not significantly different based on the current smoking status; however, chronic alcohol use increased the SDFI: 49.6 ± 23.3% (mean ± S.D.) in the semen samples from those with chronic alcohol use (*n* = 27) compared to 33.9 ± 18.0% in those who did not regularly consume alcohol (*n* = 26; *t*-test, *P* = 0.0084) (Table 3). No difference in DFI was found with regard to the prior use of medical therapy (data not shown).

If the SDFI was compared according to the conventional and computer-assisted semen parameters, the normal sperm morphology (*ρ* = −0.43883; *P* < 0.001), total sperm count (*ρ* = −0.30078; *P* = 0.02710), sperm progressive motility (*ρ* = −0.55996; *P* < 0.001), motile sperm count (*ρ* = −0.49420; *P* < 0.001), total motile sperm count (*ρ* = −0.48962; *P* < 0.001), curvilinear velocity (*ρ* = −0.26853, *P* = 0.04960), linearity (*ρ* = 0.31185; *P* = 0.02170), and amplitude of lateral head displacement (*ρ* = −0.33075; *P* = 0.01457) were related to the SDFI. The sperm count showed a trend toward being related to the SDFI (*ρ* = −0.25931; *P* = 0.05829). The SDFI was also correlated with the serum follicle-stimulating hormone level (*ρ* = 0.27100; *P* = 0.04747) but not with the serum luteinizing hormone and testosterone levels. Sperm nuclear vacuolization showed a trend to be related to the SDFI (*ρ* = 0.25796; *P* = 0.06538) (Table 4). A multivariate linear regression analysis revealed that the sperm progressive motility (*P* = 0.0008) and chronic alcohol use (*P* = 0.0394) were the significant predictive variables for sperm DNA fragmentation (Table 5).

## 4. Discussion

In the present study, sperm DNA fragmentation was evaluated in Japanese patients with male infertility. The mean SDFI was 41.3% and was greater than 30% in 32 out of the 54 subjects (59.3%). The SDFI was significantly correlated with the alcohol consumption status, serum FSH level, conventional semen parameters, and computer-assisted semen parameters. Sperm nuclear vacuolization showed a trend toward being related to the SDFI. The multivariate regression analysis revealed that chronic alcohol use and progressive sperm motility were the independent predictive variables for sperm DNA fragmentation.

Currently, there is insufficient evidence to recommend the routine use of sperm DNA integrity tests in the evaluation and treatment of infertile couples (Level C) according to the guidelines from the Practice Committee of the American Society for Reproductive Medicine [4]. However, sperm DNA damage is more common in infertile males and may contribute to poor reproductive performance. There are multiple methods to test the sperm DNA integrity [4]. The SCDt was developed and improved by Fernández et al. [12, 25]. This method is simple and easily performed in andrology laboratories and has been available in the market as the Halosperm kit. In the initial report by Fernández et al, the percentage of spermatozoa with fragmented DNA in the fertile group was 16.3 ± 6.0%, that in the normozoospermic group was 27.3 ± 11.7%, and that in the oligoasthenoteratozoospermic group was 47.3 ± 17.3%. In addition, the subject with varicocele is of clinical interest, and more than half (59.2%) of the sperm cells from the patient with varicocele contained fragmented DNA [25]. Sivanarayana et al. reported that the SDFI determined by the SCDt was as follows: 18.27 ± 7.19% in the subjects with normozoospermia, 27.56 ± 9.96% in those with teratozoospermia, 36.06 ± 11.56% in those with asthenozoospermia, and 38.15 ± 13.91% in those with oligoasthenoteratozoospermia. Therefore, the mean SDFI measured by the SCDt of 41.3% in the present study is consistent with these reports.

We used semen samples processed by a density-gradient centrifugation technique to evaluate the SDFI. However, it has not yet been determined whether processed semen samples are better than unprocessed ejaculate samples when performing the SCDt. Ebner et al. compared sperm preparation techniques in terms of the SDFI measured by the SCDt in subfertile males [30]. The mean percentage of affected spermatozoa in the ejaculate was 15.8 ± 7.8% (range 5.0–42.1%). The use of a density gradient did not significantly improve the quality of the spermatozoa selected (14.2 ± 7.0%). Simon et al. reported that the SDFI measured by the alkaline comet assay was lower in the semen processed by density gradient centrifugation [31, 32]. The SDFI in native semen showed a higher specificity and positive predictive value for predicting clinical pregnancy after IVF [32]. Therefore, it may not compromise the results to use semen samples processed by a density gradient centrifugation method when evaluating sperm DNA fragmentation.

With regard to the relationships between the SDFI measured by the SCDt and the conventional semen parameters, Velez de la Calle et al. reported that a statistically significant correlation was observed between the SDFI and semen parameters such as the sperm motility, morphology, and concentration [33]. Zhang et al. compared the SCDt with the semen parameters [34]. There were weak but significant linear relationships between the sperm concentration and sperm DNA fragmentation (*r* = − 0.272 in SCDt) and between normal morphology and sperm DNA fragmentation (*r* = − 0.283 in SCDt). The linear relationship between forward motility and sperm DNA fragmentation was moderate (*r* = − 0.477 in SCDt). In a multiple regression analysis, after controlling for the effects of the other two factors, the forward motility still maintained a negative association with the sperm DNA fragmentation.

A study by Sivanarayana et al. also reported that sperm with DNA fragmentation showed a negative correlation with semen parameters; the sperm count, motility, and normal morphology were significantly lower in the abnormal DNA group than in the normal DNA group [35]. Muriel et al. reported that the SCD was negatively correlated with the sperm motility in both ejaculated and processed semen in the setting of intrauterine insemination [36]. The results of the present study are consistent with these reports. However, this has not always been the case. In the study by Enciso et al., the proportion of degraded or fragmented spermatozoa was similar in infertile normozoospermic males (11.1 ± 9.9%) and infertile males with abnormal semen parameters (12.2 ± 8.3%) [37].

On the other hand, the relationships between the SDFI and sperm motion parameters measured by the CASA systems have not been sufficiently investigated. So far, no study has been reported that has compared the SCD and CASA-measured sperm motion characteristics. Using the comet assay, Irvine et al. reported that 56% of DNA damage was explained by the combination of the sperm concentration, spontaneous reactive oxygen species chemiluminescence, and sperm movement (ALH) in a multiple linear regression, with a positive correlation to the ALH [38]. Moskovtsev et al. reported a correlation between the sperm DNA damage measured by SCSA and the severity of semen abnormalities [39]. In those who showed an increased SDFI (≥30%), the linearity, straight-line velocity, curvilinear velocity, and ALH were significantly decreased compared to those who showed a lower SDFI (<15%). On the other hand, we found an inverse relationship with the curvilinear velocity and ALH and a positive relationship with the linearity when the SDFI was measured by SCDt in the present study. These inconsistent results may be due to the differences in the patient populations and in the methods used to measure the DNA integrity. As Feijó et al. speculated, each test for DNA integrity may identify different aspects of DNA damage [40].

Numerous vacuoles have been identified inside the sperm nucleus under light microscopes at high magnification. Many studies have indicated that there is a positive relationship between sperm DNA fragmentation (mainly measured by the TUNEL assay) and large vacuoles in the sperm nuclear area [41–44]. Therefore, the presence of sperm nuclear vacuoles on real-time optical microscopy without denaturation may indirectly indicate spermatozoa carrying possible DNA alterations. We previously reported that the percentage of spermatozoa with large nuclear vacuoles increases significantly as the semen quality decreases [5]. The proportion of spermatozoa with large nuclear vacuoles exhibits significant negative correlations with various parameters in the conventional semen and computer-assisted sperm analyses in male patients with infertility [5]. Perdrix et al. reported similar findings [45]. We used a modified classification of vacuolated spermatozoa in the present study, because the large nuclear vacuolization was not revealed to have a meaningful relationship to the SDFI (data not shown). Therefore, we included smaller vacuoles as abnormal findings (Figure 1).

In the present study, sperm nuclear vacuolization showed a nonsignificant trend toward being related to the SDFI measured by the SCDt. However, the sperm DNA fragmentation measured by the SCDt and its relationship to the observation of spermatozoa by high magnification were poorly investigated. Maettner et al. reported that the intracytoplasmic morphologically selected sperm injection (IMSI) technique alone is not sufficient for the selection of spermatozoa with intact nuclei [46]. Lopez et al. reported that their multiple logistic regression showed that DNA fragmentation, sperm vacuolization, and the number of embryos obtained per cycle are significant independent variables related to pregnancies; however, they did not analyze the relationship between the SDFI and sperm vacuolization [47]. Further investigation is needed to elucidate the relationship between the SCD and sperm nuclear vacuolization.

There is increasing acceptance that the sperm DNA fragmentation is associated with varicoceles. Several groups reported that varicoceles are associated with increased sperm DNA damage. Infertile males with varicoceles showed significantly increased sperm DNA damage, as measured by the SCSA, which appeared to be related to high levels of oxidative stress in the semen [48]. Enciso et al. reported that the varicocele patients had a more significantly increased proportion of spermatozoa with degraded or fragmented DNA measured by the SCDt than the infertile normozoospermic patients, infertile patients with abnormal semen parameters, and the fertile subjects [37]. In addition, significantly decreased percentages of spermatozoa with DNA fragmentation, as measured by the TUNEL assay (2.1 ± 0.4), were also found after subinguinal microsurgical varicocelectomy compared with the baseline values (5.0 ± 3.0%) [49]. In the study by Smit et al., the sperm parameters significantly improved and the sperm DNA fragmentation measured by the SCSA was significantly decreased after varicocelectomy [50]. Low DNA fragmentation index values are associated with a higher pregnancy rate (spontaneous and with assisted reproductive techniques). Therefore, sperm DNA fragmentation is substantially related to infertility in patients with a clinical varicocele.

Regarding the patients with idiopathic causes of infertility, one cannot ignore the relationship with sperm DNA fragmentation. A significant number of patients diagnosed with unexplained (normozoospermic) infertility according to traditional diagnostic methods had remarkably high degrees of fragmented sperm DNA [51]. Zinc, D-aspartate, and coenzyme Q10 may be useful as antioxidant therapy for male infertility patients without varicoceles, and a previous study showed that they exerted a direct protective effect on human spermatozoa, preventing the decrease in motility and the increase in DNA fragmentation measured by the TUNEL assay during* in vitro* culture [52]. Abad et al. analyzed the effects of antioxidant therapy for sperm DNA fragmentation [53]. The proportion of highly DNA degraded sperm was also significantly reduced in their study (*P* < 0.05). A semen analysis showed that there was a significant increase in the concentration, motility, vitality, and morphology parameters, with a decrease in sperm DNA fragmentation measured by the SCDt. These findings could explain the fact that no difference was found in the SDFI based on the causes of male infertility in the present study. In the present study, eight patients underwent medical therapy by Japanese herbal medicines; however, there was not significant difference in the SDFI as compared to those without medical therapy. We cannot decide the influence of this kind of medicine on the SDFI due to the limited number of patients.

Among the modifiable lifestyle factors, smoking is thought to induce sperm DNA damage and cause poor semen quality [17]. Germinal cells are vulnerable to genetic damage from smoking [18]. However, we did not find any significant effect of smoking on the sperm DNA fragmentation. This may be due to the limited number of semen samples from current smokers (*n* = 10) in our cohort, and our finding regarding smoking is not conclusive.

Possible associations between alcohol exposure and reduced male fertility have also been the subjects of various studies [19]. Experimental and clinical studies have suggested that alcohol consumption may alter both testosterone secretion and spermatogenesis. Fas system upregulation and elevated caspase activity in the testes of ethanol-treated rats may be a reflection of ethanol-induced testicular injury resulting in enhanced germ cells apoptosis, which may be involved in the infertility associated with alcohol abuse [54]. Sperm DNA integrity is also reported to be influenced by alcohol exposure. In an adult Wistar rat model, ethanol consumption disturbs sperm motility, nuclear maturity, and DNA integrity of spermatozoa. Ethanol abuse results in the production of spermatozoa with less condensed chromatin, and this may be one possible cause of infertility following ethanol consumption [23]. It is reported that alcohol has negative effects on sperm parameters, chromatin/DNA integrity, and apoptosis in mice [24]. From human studies, it is known that alcohol consumption produces significant morphological changes in the spermatozoa, which include breakage of the sperm head, distention of the midsection, and tail curling [22, 55]. In addition, the seminiferous tubules in alcohol users mostly contain degenerated spermatids, with a consequent azoospermia [22].

These effects may be due to alterations of the endocrine system controlling the hypothalamic-pituitary-testicular (HPT) axis function and/or to a direct effect on testis and/or male accessory glands [20–22]. Ethanol is transferred to the male reproductive tract and has a toxic effect on Leydig cells, influencing the testosterone level, as well as the Leydig cell volume [56, 57]. An association between increased alcohol intake and decreased spermatogenesis was reported in human autopsy studies [20, 58]. One* in vitro* study by Donnelly et al. showed that damaging effects were observed in both sperm motility and morphology when alcohol was added directly to sperm, at concentrations equivalent to those in serum after moderate and heavy drinking [59]. Hansen et al. reported an association between recent alcohol intake and a hormonal shift towards a higher estradiol/testosterone ratio [19].

In the present study, the serum FSH level was higher in those with chronic alcohol use (7.9 ± 0.8 mIU/mL) than in those without regular alcohol consumption (5.7 ± 0.5 mIU/mL). Total sperm count was relatively lower in those with chronic alcohol use than in those without although it was not significant (109.1 ± 24.2 million versus 174.2 ± 24.7 million, resp.; *P* = 0.0656). Therefore, chronic alcohol use impaired the spermatogenesis to some extent in our cohort. In addition, chronic alcohol use is an independent factor associated with increased DFI.

There are some limitations associated with this study. First, the number of subjects was relatively small. Second, the sperm DNA fragmentation was evaluated only by using the SCDt. Third, occupational and educational factors were not collected. If the study was conducted using a larger cohort and other methods for sperm DNA fragmentation, the results may be different. The pregnancy status was not analyzed in this cohort. However, only two couples had obtained pregnancy by the end of this study; one obtained pregnancy by natural conception after the administration of an oriental herbal medicine for idiopathic male infertility (SDFI = 13.9%) and the other obtained pregnancy by intracytoplasmic sperm injection (varicocele, SDFI = 66.7). Therefore, we cannot analyze the data according to the pregnancy rates.

## 5. Conclusions

In conclusion, the mean SDFI was 41.3% in Japanese patients with male infertility, regardless of its cause. If 22.75% is used as a threshold value for infertility, 41 out of the 54 (75.9%) subjects in our study had increased sperm DNA fragmentation [16]. The SDFI was related to a poor semen quality, as measured by conventional semen parameters, sperm motion characteristics measured by the computer-assisted semen analyses, and chronic alcohol use. The multivariate linear regression analysis revealed that sperm progressive motility and chronic alcohol use were the independent variables that predicted sperm DNA fragmentation. Sperm DNA integrity testing may be offered to those who regularly consume alcohol or who have decreased sperm progressive motility. Further studies are needed to confirm the present results.

## Figures and Tables

**Table 1:** The characteristics of the patients who underwent semen analyses (*n* = 54).

Parameter	Mean	S.D.	Median	Interquartile range
Age (years old)	34.9	5.1	35	31–39
Age of female partner (years old)	33.2	4.6	32	30–37
Duration of infertility (months)	36.0	28.3	30	17.3–43.3
Serum LH (mIU/mL)	4.80	2.34	4.20	3.28–5.85
Serum FSH (mIU/mL)	6.73	3.54	5.6	4.40–8.63
Serum total testosterone (ng/mL)	4.80	1.59	4.84	3.44–5.93
Serum free testosterone (pg/mL)	9.56	2.85	9.25	7.6–11.3
Brinkman index^#^	138	236	0	0–235
Body mass index (kg/cm^2^)	23.5	2.7	23.3	21.3–26.9

Causes of male infertility			
Idiopathic		*n* = 21	
Varicocele		*n* = 33		
Chronic alcohol use			
(+)		*n* = 27	
(−)		*n* = 27		
Current smoking			
(+)		*n* = 10	
(−)		*n* = 43	
Unknown		*n* = 1		

S.D.: standard deviation; LH: luteinizing hormone; FSH: follicle-stimulating hormone; ^#^daily number of cigarettes × years.

**Table 2:** The results of the conventional semen analyses, computer-assisted semen analyses, high-magnification observations of spermatozoa, and SCD tests.

Parameter	Mean	S.D.	Median	Interquartile range
Conventional semen analyses (*n* = 54)				
Abstinence (Days)	5.8	2.5	5.0	5.0-6.0
Semen volume (mL)	3.1	1.6	3.0	2.0–5.2
Normal sperm morphology (%)	2.1	2.1	1.5	0.6–3.0
Sperm count (10^6^/mL)	49.8	52.8	35.7	17.8–61.0
Total sperm count (10^6^)	146.1	133.1	97.0	38.7–227.9
Progressive sperm motility (%)	33.3	17.9	33.0	19.5–47.3
Motile sperm count (10^6^/mL)	22.0	43.4	8.2	3.1–25.6
Total motile sperm count (10^6^)	57.0	81.2	22.9	7.7–99.4
Computer-assisted semen analyses (SMAS, *n* = 54)				
Linear velocity (*μ*m/sec)	17.8	4.8	17.7	15.5–21.6
Curvilinear velocity (*μ*m/sec)	45.0	12.4	46.4	36.2–52.4
Linearity	0.40	0.08	0.40	0.36–0.45
ALH (*μ*m)	1.048	0.362	1.045	0.76–1.335
Beat/cross frequency (Hz)	11.19	1.84	11.22	10.60–12.19
High-magnification microscopy (*n* = 52)				
Proportion of vacuolated sperm (%)	24.7	17.9	19.0	11.5–36.4
SCD tests (*n* = 54)				
Sperm DNA fragmentation index (%)	41.3	22.3	42.7	22.5–56.2

S.D.: standard deviation; SMAS: sperm motility analysis system; ALH: amplitude of lateral head displacement; SCD test: sperm chromatin dispersion test.

**Table 3:** The correlations among the sperm DFI and causes of male infertility and regular alcohol intake and smoking status.

Parameter	DFI (%, mean ± S.D.)	*P* values
Cause of male infertility		
Idiopathic (*n* = 21)	41.3 ± 21.9	0.9963
Varicocele (*n* = 33)	41.3 ± 22.9	
Chronic alcohol use		
(+) (*n* = 27)	49.6 ± 23.3	0.0084∗
(−) (*n* = 27)	33.9 ± 18.0	
Current smoking		
(+) (*n* = 10)	51.3 ± 21.8	0.1357
(−) (*n* = 43)	39.7 ± 21.9	

DFI: DNA fragmentation index; S.D.: standard deviation; *statistically significant (Student's *t*-test).

**Table 4:** The correlations between the sperm DNA fragmentation index and the clinical parameters.

Variable	*ρ*	*P* values
Age (years old)	0.09448	0.49678
Duration of infertility (months)	0.15084	0.27626
Serum LH (mIU/mL)	0.09629	0.48855
Serum FSH (mIU/mL)	0.27100	0.04747∗
Serum total testosterone (ng/mL)	0.07281	0.60082
Serum free testosterone (pg/mL)	0.05565	0.69519
Brinkman index^#^	0.10040	0.47442
Body mass index (kg/m^2^)	0.17027	0.21835
Conventional semen analysis		
Abstinence (days)	−0.12276	0.37651
Semen volume (mL)	−0.10769	0.43829
Normal sperm morphology (%)	−0.43883	0.00090∗
Sperm count (10^6^/mL)	−0.25931	0.05829
Total sperm count (10^6^)	−0.30078	0.02710
Sperm progressive motility (%)	−0.55996	0.00001∗
Motile sperm count (10^6^/mL)	−0.49420	0.00015∗
Total motile sperm count (10^6^)	−0.48962	0.00017∗
Computer-assisted semen analysis		
Linear velocity (*μ*m/sec)	−0.01841	0.89488
Curvilinear velocity (*μ*m/sec)	−0.26853	0.04960∗
Linearity	0.31185	0.02170∗
Amplitude of lateral head displacement (*μ*m)	−0.33075	0.01457∗
Beat/cross frequency (Hz)	−0.14489	0.29587
High-magnification microscopy		
Proportion of vacuolated sperm (%)	0.25746	0.06538

LH: luteinizing hormone; *ρ*: Spearman's rank correlation coefficient; FSH: follicle-stimulating hormone; *statistically significant; ^#^daily number of cigarettes × years.

**Table 5:** The attributes of the clinical parameters associated with DNA damage in the multiple linear regression.

Parameter	Standardized partial regression coefficient	*P* value	95% confidence interval	
			Lower limit	Upper limit
Model 1				
Chronic alcohol use (+) versus (−)	0.2744	0.0394∗	0.2922	11.1788
Serum FSH ≥5.6 versus <5.6 (mIU/mL)	0.1429	0.2466	−2.1513	8.1519
Normal sperm morphology ≥1.5 versus <1.5 (%)	−0.0557	0.6813	−6.8560	4.5226
Sperm progressive motility ≥33.0 versus <33.0 (%)	−0.4864	0.0008∗	−15.9232	−4.5046
Total sperm count ≥97.0 versus <97.0 (million)	−0.0605	0.6369	−6.6348	4.1036
Linearity ≥0.4 versus <0.4	−0.0471	0.7221	−6.5417	4.5696
Proportion of vacuolated sperm ≥19.0 versus 19.0 (%)	0.0669	0.5782	−3.6395	6.4395
Model 2				
Chronic alcohol use (+) versus (−)	0.3069	0.0068∗	1.9444	11.5084
Sperm progressive motility ≥33.0 versus <33.0 (%)	−0.5381	<0.0001∗	−16.5970	−7.0193

ALH: amplitude of lateral head displacement; FSH: follicle-stimulating hormone; *statistically significant.

## References

[1] Guzick DS, Overstreet JW, Factor-Litvak P (2001). Sperm morphology, motility, and concentration in fertile and infertile men. *The New England Journal of Medicine*.

[2] Jedrzejczak P, Taszarek-Hauke G, Hauke J, Pawelczyk L, Duleba AJ (2008). Prediction of spontaneous conception based on semen parameters. *International Journal of Andrology*.

[3] Akashi T, Watanabe A, Komiya A, Fuse H (2010). Evaluation of the Sperm Motility Analyzer System (SMAS) for the assessment of sperm quality in infertile men. *Systems Biology in Reproductive Medicine*.

[4] The Practice Committee of the American Society for Reproductive Medicine (2013). The clinical utility of sperm DNA integrity testing: a guideline. *Fertility and Sterility*.

[5] Komiya A, Watanabe A, Kawauchi Y, Fuse H (2013). Sperm with large nuclear vacuoles and semen quality in the evaluation of male infertility. *Systems Biology in Reproductive Medicine*.

[6] Setti AS, Paes de Almeida Ferreira Braga D, Iaconelli A, Aoki T, Borges E (2013). Twelve years of MSOME and IMSI: a review. *Reproductive BioMedicine Online*.

[7] Perdrix A, Rives N (2013). Motile sperm organelle morphology examination (MSOME) and sperm head vacuoles: state of the art in 2013. *Human Reproduction Update*.

[8] Agarwal A, Said TM (2003). Role of sperm chromatin abnormalities and DNA damage in male infertility. *Human Reproduction Update*.

[9] Evenson DP, Jost LK, Marshall D (1999). Utility of the sperm chromatin structure assay as a diagnostic and prognostic tool in the human fertility clinic. *Human Reproduction*.

[10] Sun JG, Jurisicova A, Casper RF (1997). Detection of deoxyribonucleic acid fragmentation in human sperm: correlation with fertilization in vitro. *Biology of Reproduction*.

[11] Singh NP, McCoy MT, Tice RR, Schneider EL (1988). A simple technique for quantitation of low levels of DNA damage in individual cells. *Experimental Cell Research*.

[12] Fernández JL, Muriel L, Rivero MT, Goyanes V, Vazquez R, Alvarez JG (2003). The sperm chromatin dispersion test: a simple method for the determination of sperm DNA fragmentation. *Journal of Andrology*.

[13] Collins JA, Barnhart KT, Schlegel PN (2008). Do sperm DNA integrity tests predict pregnancy with in vitro fertilization?. *Fertility and Sterility*.

[14] Zini A, Boman JM, Belzile E, Ciampi A (2008). Sperm DNA damage is associated with an increased risk of pregnancy loss after IVF and ICSI: systematic review and meta-analysis. *Human Reproduction*.

[15] Wang Y-J, Zhang R-Q, Lin Y-J, Zhang R-G, Zhang W-L (2012). Relationship between varicocele and sperm DNA damage and the effect of varicocele repair: a meta-analysis. *Reproductive BioMedicine Online*.

[16] Ribas-Maynou J, García-Peiró A, Fernández-Encinas A (2013). Comprehensive analysis of sperm DNA fragmentation by five different assays: TUNEL assay, SCSA, SCD test and alkaline and neutral Comet assay. *Andrology*.

[17] Shen HM, Chia SE, Ong CN (1999). Evaluation of oxidative DNA damage in human sperm and its association with male infertility. *Journal of Andrology*.

[18] Zenzes MT (2000). Smoking and reproduction: gene damage to human gametes and embryos. *Human Reproduction Update*.

[19] Hansen ML, Thulstrup AM, Bonde JP, Olsen J, Håkonsen LB, Ramlau-Hansen CH (2012). Does last week’s alcohol intake affect semen quality or reproductive hormones? A cross-sectional study among healthy young Danish men. *Reproductive Toxicology*.

[20] Kuller LH, May SJ, Perper JA (1978). The relationship between alcohol, liver disease, and testicular pathology. *The American Journal of Epidemiology*.

[21] Anderson RA, Willis BR, Oswald C, Zaneveld LJD (1985). Partial reversal of ethanol-induced male reproductive pathology following abstinence. *Alcohol and Alcoholism*.

[22] Hadi HA, Hill JA, Castillo RA (1987). Alcohol and reproductive function: a review. *Obstetrical and Gynecological Survey*.

[23] Talebi AR, Sarcheshmeh AA, Khalili MA, Tabibnejad N (2011). Effects of ethanol consumption on chromatin condensation and DNA integrity of epididymal spermatozoa in rat. *Alcohol*.

[24] Rahimipour M, Talebi AR, Anvari M, Sarcheshmeh AA, Omidi M (2013). Effects of different doses of ethanol on sperm parameters, chromatin structure and apoptosis in adult mice. *European Journal of Obstetrics & Gynecology and Reproductive Biology*.

[25] Fernández JL, Muriel L, Goyanes V (2005). Simple determination of human sperm DNA fragmentation with an improved sperm chromatin dispersion test. *Fertility and Sterility*.

[26] WHO (1999). *World Health Organization Laboratory Manual for the Examination of Human Semen and Sperm-Cervical Mucus Interaction*.

[27] Fuse H, Iwasaki M, Mizuno I, Ikehara-Kawauchi Y (2003). Evaluation of acrosome reactivity using the Acrobeads test in varicocele patients: findings before and after treatment. *Systems Biology in Reproductive Medicine*.

[28] Bartoov B, Berkovitz A, Eltes F, Kogosowski A, Menezo Y, Barak Y (2002). Real-time fine morphology of motile human sperm cells is associated with IVF-ICSI outcome. *Journal of Andrology*.

[29] Bartoov B, Berkovitz A, Eltes F (2003). Pregnancy rates are higher with intracytoplasmic morphologically selected sperm injection than with conventional intracytoplasmic injection. *Fertility and Sterility*.

[30] Ebner T, Shebl O, Moser M, Mayer RB, Arzt W, Tews G (2011). Easy sperm processing technique allowing exclusive accumulation and later usage of DNA-strandbreak-free spermatozoa. *Reproductive BioMedicine Online*.

[31] Simon L, Lewis SEM (2011). Sperm DNA damage or progressive motility: which one is the better predictor of fertilization in vitro?. *Systems Biology in Reproductive Medicine*.

[32] Simon L, Lutton D, McManus J, Lewis SEM (2011). Sperm DNA damage measured by the alkaline Comet assay as an independent predictor of male infertility and in vitro fertilization success. *Fertility and Sterility*.

[33] Velez de la Calle JF, Muller A, Walschaerts M (2008). Sperm deoxyribonucleic acid fragmentation as assessed by the sperm chromatin dispersion test in assisted reproductive technology programs: results of a large prospective multicenter study. *Fertility and Sterility*.

[34] Zhang LH, Qiu Y, Wang KH, Wang Q, Tao G, Wang LG (2010). Measurement of sperm DNA fragmentation using bright-field microscopy: comparison between sperm chromatin dispersion test and terminal uridine nick-end labeling assay. *Fertility and Sterility*.

[35] Sivanarayana T, Ravi Krishna C, Jaya Prakash G (2014). Sperm DNA fragmentation assay by sperm chromatin dispersion (SCD): correlation between DNA fragmentation and outcome of intracytoplasmic sperm injection. *Reproductive Medicine and Biology*.

[36] Muriel L, Meseguer M, Fernández JL (2006). Value of the sperm chromatin dispersion test in predicting pregnancy outcome in intrauterine insemination: a blind prospective study. *Human Reproduction*.

[37] Enciso M, Muriel L, Fernández JL (2006). Infertile men with varicocele show a high relative proportion of sperm cells with intense nuclear damage level, evidenced by the sperm chromatin dispersion test. *Journal of Andrology*.

[38] Irvine DS, Twigg JP, Gordon EL, Fulton N, Milne PA, Aitken RJ (2000). DNA integrity in human spermatozoa: relationships with semen quality. *Journal of Andrology*.

[39] Moskovtsev SI, Willis J, White J, Mullen JBM (2009). Sperm DNA damage: correlation to severity of semen abnormalities. *Urology*.

[40] Feijó CM, Esteves SC (2014). Diagnostic accuracy of sperm chromatin dispersion test to evaluate sperm deoxyribonucleic acid damage in men with unexplained infertility. *Fertility and Sterility*.

[41] Oliveira JBA, Massaro FC, Baruffi RLR (2010). Correlation between semen analysis by motile sperm organelle morphology examination and sperm DNA damage. *Fertility and Sterility*.

[42] Sakkas D, Alvarez JG (2010). Sperm DNA fragmentation: mechanisms of origin, impact on reproductive outcome, and analysis. *Fertility and Sterility*.

[43] Skowronek F, Casanova G, Alciaturi J (2012). DNA sperm damage correlates with nuclear ultrastructural sperm defects interatozoospermic men. *Andrologia*.

[44] Utsuno H, Oka K, Yamamoto A, Shiozawa T (2013). Evaluation of sperm head shape at high magnification revealed correlation of sperm DNA fragmentation with aberrant head ellipticity and angularity. *Fertility and Sterility*.

[45] Perdrix A, Saïdi R, Ménard JF (2012). Relationship between conventional sperm parameters and motile sperm organelle morphology examination (MSOME). *International Journal of Andrology*.

[46] Maettner R, Sterzik K, Isachenko V (2014). Quality of human spermatozoa: relationship between high-magnification sperm morphology and DNA integrity. *Andrologia*.

[47] Lopez G, Lafuente R, Checa MA, Carreras R, Brassesco M (2013). Diagnostic value of sperm DNA fragmentation and sperm high-magnification for predicting outcome of assisted reproduction treatment. *Asian Journal of Andrology*.

[48] Saleh RA, Agarwal A, Sharma RK, Said TM, Sikka SC, Thomas AJ (2003). Evaluation of nuclear DNA damage in spermatozoa from infertile men with varicocele. *Fertility and Sterility*.

[49] Li F, Yamaguchi K, Okada K (2012). Significant improvement of sperm DNA quality after microsurgical repair of varicocele. *Systems Biology in Reproductive Medicine*.

[50] Smit M, Romijn JC, Wildhagen MF, Veldhoven JLM, Weber RFA, Dohle GR (2013). Decreased sperm DNA fragmentation after surgical varicocelectomy is associated with increased pregnancy rate. *The Journal of Urology*.

[51] Oleszczuk K, Augustinsson L, Bayat N, Giwercman A, Bungum M (2013). Prevalence of high DNA fragmentation index in male partners of unexplained infertile couples. *Andrology*.

[52] Talevi R, Barbato V, Fiorentino I, Braun S, Longobardi S, Gualtieri R (2013). Protective effects of in vitro treatment with zinc, d-aspartate and coenzyme q10 on human sperm motility, lipid peroxidation and DNA fragmentation. *Reproductive Biology and Endocrinology*.

[53] Abad C, Amengual MJ, Gosálvez J (2013). Effects of oral antioxidant treatment upon the dynamics of human sperm DNA fragmentation and subpopulations of sperm with highly degraded DNA. *Andrologia*.

[54] Eid NAS, Shibata M, Ito Y, Kusakabe K, Hammad H, Otsuki Y (2002). Involvement of Fas system and active caspases in apoptotic signalling in testicular germ cells of ethanol-treated rats. *International Journal of Andrology*.

[55] La Vignera S, Condorelli RA, Balercia G, Vicari E, Calogero AE (2013). Does alcohol have any effect on male reproductive function? A review of literature. *Asian Journal of Andrology*.

[56] van Thiel DH, Gavaler JS, Cobb CF, Santucci L, Graham TO (1983). Ethanol, a Leydig cell toxin: evidence obtained in vivo and in vitro. *Pharmacology, Biochemistry and Behavior*.

[57] Salonen I, Eriksson CJP (1989). Penetration of ethanol into the male reproductive tract. *Alcoholism: Clinical and Experimental Research*.

[58] Pajarinen J, Karhunen PJ, Savolainen V, Lalu K, Penttilä A, Laippala P (1996). Moderate alcohol consumption and disorders of human spermatogenesis. *Alcoholism: Clinical and Experimental Research*.

[59] Donnelly GP, McClure N, Kennedy MS, Lewis SEM (1999). Direct effect of alcohol on the motility and morphology of human spermatozoa. *Andrologia*.

